# Fabrication and Luminescent Properties of Highly Transparent Er:Y_2_O_3_ Ceramics by Hot Pressing Sintering

**DOI:** 10.3390/ma16134504

**Published:** 2023-06-21

**Authors:** Yan Liu, Chengrui Liu, Xianpeng Qin, Lin Gan, Guohong Zhou, Juan Jiang, Tianjin Zhang, Hetuo Chen, Zhengjuan Wang, Shiwei Wang

**Affiliations:** 1 State Key Laboratory of High Performance Ceramics and Superfine Microstructure, Shanghai Institute of Ceramics, Chinese Academy of Sciences, Shanghai 200050, China; ly8267426@163.com (Y.L.); liuchengrui961213@163.com (C.L.); sic_zhough@mail.sic.ac.cn (G.Z.); chenhetuo@mail.sic.ac.cn (H.C.); wzhj926@mail.sic.ac.cn (Z.W.);; 2 Collaborative Innovation Center for Advanced Organic Chemical Materials Co-Constructed by the Province and Ministry, Ministry of Education Key Laboratory for the Green Preparation and Application of Functional Materials, School of Material Science and Engineering, Hubei University, Wuhan 430062, China; jiangjuan@hubu.edu.cn (J.J.); zhangtj@hubu.edu.cn (T.Z.); 3 Center of Materials Science and Optoelectronics Engineering, University of Chinese Academy of Sciences, Beijing 100049, China; 4 Suzhou Research Institute, Shanghai Institute of Ceramics, Chinese Academy of Sciences, Taicang 215400, China

**Keywords:** highly transparent, submicro-grained Er:Y_2_O_3_, luminescent, hot pressing method

## Abstract

Highly transparent Er:Y_2_O_3_ ceramics (1–9 at.% Er) were fabricated by hot pressing sintering with ZrO_2_ as the sintering additive. The microstructures, transmittance, luminescent properties, thermal conductivity, and mechanical properties of the Er:Y_2_O_3_ ceramic samples were investigated in detail. The samples all exhibited dense and fine grain microstructures; the average grain sizes were about 0.8 μm. The transmittance levels of the samples with various Er concentrations (2 mm thick) at the wavelengths of 600 and 2700 nm were ~74 and ~83%, respectively. As the Er doping concentration increased from 1 to 9 at.%, the up-conversion luminescence of the samples gradually changed from green to red, with the intensity ratio of red/green light increasing from 0.28 to 2.01. Meanwhile, the down-conversion luminescence properties of the specimens were also studied. When the samples were under 980 nm excitation, the emission bands were detected at 1552, 1573, 1639, and 1661 nm. The thermal conductivity of the samples was found to decrease from 8.72 to 5.81 W/(m·K) with an increase of the Er concentration from 1 to 9 at.%. Moreover, the microhardness and fracture toughness of the samples with 1 at.% Er concentration were ~8.51 GPa and ~1.03 MPa·m^1/2^, respectively.

## 1. Introduction

In recent decades, great efforts have focused on the development of rare earth (RE)-based transparent polycrystalline materials, such as Y_3_Al_5_O_12_ (YAG) and RE_2_O_3_ (RE = Y, Sc, or Lu), because of their potential applications as solid-state laser hosts, optical windows, and scintillators [1]. Compared to the commonly used YAG laser materials (the phonon energy is 875 cm^−1^, thermal conductivity is ~11 W/(m·K)), RE_2_O_3_ compounds have lower phonon energy levels (i.e., 565, 672, and 612 cm^−1^ for Y_2_O_3_, Sc_2_O_3_, and Lu_2_O_3_, respectively) and higher thermal conductivities (i.e., ~17, ~16.5, and ~12.5 W/(m·K) for Y_2_O_3_, Sc_2_O_3_, and Lu_2_O_3_) [2,3,4,5]. Apparently, Y_2_O_3_ possesses the lowest phonon energy level, which is beneficial for suppressing the multi-phonon non-radiative quenching. Meanwhile, Y_2_O_3_ (~17 W/m·K) [4] has a higher thermal conductivity compared to YAG (~11 W/m·K) [5], which is quite favorable for the thermal management of laser gain media. Therefore, transparent polycrystalline Y_2_O_3_ is widely considered a promising laser host material.

A series of RE-doped (e.g., Nd^3+^, Yb^3+^, Ho^3+^, Er^3+^, etc.) transparent Y_2_O_3_ ceramic materials have been successfully developed [6,7,8]. Among them, Er-doped Y_2_O_3_ showed more complicated luminescent behavior and therefore have greater potential applications [9,10,11,12,13,14,15,16,17]. When excited by commercially available 980 nm laser diodes (LD), Er-doped Y_2_O_3_ gives both up- and down-conversion emissions. The up-conversion luminescence is attributed to the ^2^H_11/2_/^4^S_3/2_ → ^4^I_15/2_ (~525–575 nm) and ^4^F_9/2_ → ^4^I_15/2_ (~650–680 nm) transitions, while the down-conversion one is due to the ^4^I_13/2_ → ^4^I_15/2_ (~1400–1700 nm) transition [9,14]. Compared to Nd^3+^ or Yb^3+^ ions, Er^3+^ in the cubic Y_2_O_3_ lattice shows down-conversion luminescence at longer wavelengths (i.e., ~1 μm for Nd^3+^ and Yb^3+^, 1.4–1.7 μm for Er^3+^), which makes Er-doped Y_2_O_3_ an excellent candidate material for eye-safe solid-state lasers [6,7,8,9].

However, it is not easy to fabricate transparent Y_2_O_3_ ceramics because of the high melting point of yttria (i.e., 2430 °C). According to the available literature, previously reported Er:Y_2_O_3_ transparent ceramics were fabricated by either a vacuum sintering method [18] or a hot-isostatic pressing route (HIP) [19]. The former method is very energy-intensive, requiring high sintering temperatures (e.g., ≥1800 °C) and long holding times (e.g., ~16 h). While the latter is quite uneconomical as a large amount of inert gas has to be used in the HIP process. In this work, highly transparent Y_2_O_3_ ceramics with various Er concentrations were fabricated by a convenient and economical hot-pressing method. The optical transmittance, microstructures, luminescence, and thermo-mechanical properties of the samples were investigated with respect to solid laser applications.

## 2. Materials and Methods

High-purity powders of Y_2_O_3_ (99.99%, Jiangyin Jiahua, China), Er_2_O_3_(99.995%, Rare-Chem, Huizhou, China), and Zr(NO_3_)_4_·3H_2_O (AR, Shanghai Diyang, Shanghai, China) powders were used as starting materials. According to the compositions of Y_(1_._98−x)_Zr_0_._02_Er_x_O_3_ (x = 0.02, 0.06, 0.10, 0.14, and 0.18, the concentration of Er is 1, 3, 5, 7, and 9 at.%, respectively), the powders were weighed and milled by ball milling with 3 mm ZrO_2_ balls in anhydrous ethanol at 250 rpm for 24 h (the mass ratio of powder, alcohol, and ball is 1:2:5). Next, the powder mixtures were dried at 60 °C for 24 h, the powders were sieved through a 200-mesh screen, then were calcined at 1200 °C for 4 h to remove any organic component completely. The calcined powders were dry pressed in a stainless-steel mold at 5 MPa. The green bodies were pre-sintered at 1400 °C for 2 h in a muffle furnace to enhance their strength. The pre-sintered bodies were wrapped with tantalum foil and hot-pressed at 1600 °C for 3 h at a mechanical pressure of 20 MPa under a vacuum of ~10^−3^ Pa. After the sintering step, the samples were annealed at 1400 °C for 5 h in air. Finally, all the sintered samples were double-side polished to a thickness of 2 mm for measurements.

Phase identification was carried out by an X-ray diffraction (XRD, D/max 2550 V, JPAT, Tokyo, Japan) analysis using Cu Kα radiation. According to the XRD data, the lattice parameter and unit cell volume of the ceramic samples were calculated. Based on the calculated unit cell volume, the theoretical densities of the samples were calculated using Equation (1) [20]:

*ρ*_*th*_ = *ZM*/*NV* (1)

where *Z* is the number of molecules in a unit cell (*Z* = 16), *M* is the molecular weight, *N* is the Avogadro’s constant, and *V* is the unit cell volume. By the Archimedes method, the bulk densities of the samples were measured. The ratio between each specimen’s bulk density and theoretical density was used to calculate the relative densities. The microstructures of the thermal etched surfaces and the fracture surfaces of the transparent ceramics were observed by scanning electron microscope (SEM, TM-3000, HITACHI, Tokyo, Japan). Using the SEM images from the polished and thermally etched surfaces, the Nano Measurer (1.2) software calculated the average grain sizes of the samples. Optical transmittance and absorbance spectra of the samples were measured by a UV–VIS–NIR (V770, JASCO, Tokyo, Japan) spectrometer in the range from 190 to 2700 nm. Fluorescence and up-conversion luminescence spectra were measured on the ceramics at room temperature by spectrofluorometer (FLS-980, Edinburgh, UK), and a 980 nm continuous wave laser diode was used as the excitation. A Vickers hardness tester (HVS-5Z/LCD, Shanghai Taming Optical Instrument Co., Ltd., Shanghai, China) with five indentations was used to assess the microhardness and fracture toughness. The microhardness (*H*, GPa) was calculated using Equation (2):

*H* = 4*kP*/(*d*_1_ + *d*_2_)^2^ (2)

where *P* is the load (9.8 N) on the indenter, *d*_1_ and *d*_2_ are the indentation diagonals, and *k* is the shape factor of the indenter, which is 1.8544. The fracture toughness was determined by measuring the lengths of the cracks. The fracture toughness (*K*_Ic_, MPa·m^1/2^) was calculated using Equation (3) [21]:

*K*_Ic_ = 9.81 × 1000 × *P*(3.14*c*)^−3/2^/[tan(68 × 3.14/180)]^−1^ (3)

where *P* is the load (9.8 N) on the indenter and *c* is the average lengths of the cracks. The thermal diffusivity and heat capacity of the ceramic specimens were measured using a laser flash analyzer (LFA467, Netzsch, Serb, Germany). The thermal conductivity was calculated using Equation (4):

*k* = *α*·*ρ*·*c*_p_ (4)

where *k* (W/(m·K)) signifies the thermal conductivity, *α* denotes the thermal diffusivity, *ρ* represents the density, and *c*_p_ is the heat capacity.

## 3. Results and Discussion

**Figure 1:**
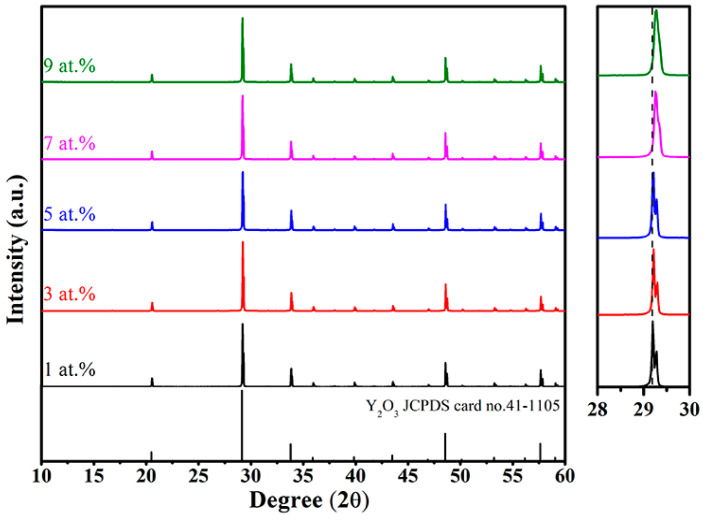
XRD patterns of the Er:Y_2_O_3_ ceramics with various Er concentrations.

Figure 1 displays the XRD patterns of the samples sintered at 1600 °C for 3 h with various Er concentrations. All the observed peaks matched well with the cubic yttria phase (JCPDS: 41-1109), and no apparent impurity phase was observed. This implies that the Zr^4+^ and Er^3+^ ions were incorporated into the yttria lattice. It was also found that the diffraction peaks of the Er:Y_2_O_3_ ceramics gradually shifted to higher angles with an increase in the Er concentration (see the right inset in Figure 1), which is ascribed to the shrinkage of the lattice of yttria caused by the partial substitution of smaller Er^3+^ (0.89 Å (CN = 6)) for larger Y^3+^ (0.90 Å (CN = 6)) [22].

Table 1 exhibits the calculated structural parameters (i.e., lattice parameters and unit cell volume) and densities (i.e., theoretical density (*ρ_th_*), bulk density (*ρ_ap_*), and relative density (*ρ_re_*)) of the samples. Both of the lattice parameters and unit cell volume decreased with an increase in the Er concentration, which is consistence with the XRD results. Furthermore, as the Er concentration increased, the theoretical density of the samples also increased as a result of the lattice shrinkage and the larger atomic weight of Er compared to that of Y. While the relative density was not strongly influenced by the doping concentration, all samples possessed high levels (i.e., ~99.9%) of the relative density.

**Figure 2:**
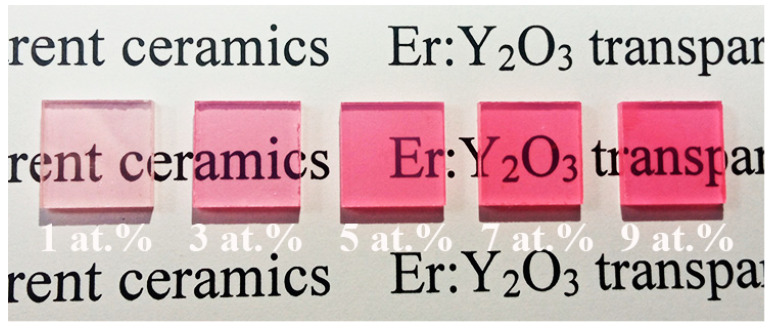
Photographs of the Er:Y_2_O_3_ ceramic samples with various Er concentrations (2 mm thick).

Figure 2 shows photographs of the Er:Y_2_O_3_ ceramics with various Er concentrations. Apparently the specimens were highly transparent, as the words below them can be clearly seen. In addition, the samples showed a reddish color, and the color became deeper with an increase in the Er doping concentration.

**Figure 3:**
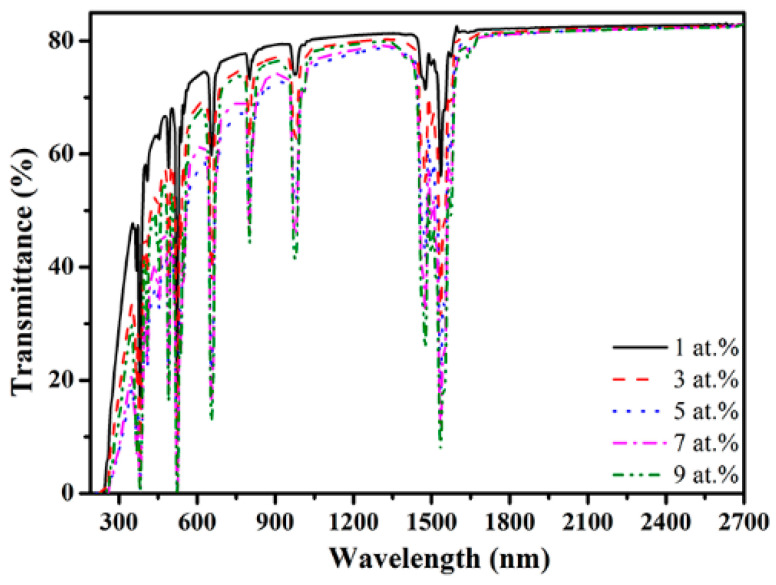
In-line transmittance of the samples with various Er concentrations.

Figure 3 presents the in-line transmittance of the samples with various Er concentrations. The hot-pressed Er:Y_2_O_3_ ceramics exhibited excellent optical transparency in the near-infrared and visible regions. For the sample doped with 1 at.% Er, the transmittance rates at the wavelengths of 600 and 2700 nm were 73.7 and 82.8%, respectively. These transmittance levels are comparable to those of the Yb:Y_2_O_3_ transparent ceramics by hot pressing in our previous work [23]. Additionally, the specimens with 1, 3, and 9 at.% Er showed higher transmittance compared to the samples with 5 and 7 at.% Er, this may be due to the fact that the samples with 1, 3, and 9 at.% Er exhibited slightly higher relative densities compared to the samples with 5 and 7 at.% Er as shown in Table 1. Residual pores are known as strong light-scattering centers, which can remarkably decrease the optical transparency of transparent ceramic materials [18]. Furthermore, the absorption bands centered at 382, 525, 654, 800, 972, and 1535 nm are attributed to the transitions of Er^3+^ ions from the ground state of ^4^I_15/2_ to the excited states of ^4^G_11/2_, ^2^H_11/2_, ^4^F_9/2_, ^4^I_9/2_, ^4^I_11/2_, and ^4^I_13/2_, respectively [24].

**Figure 4:**
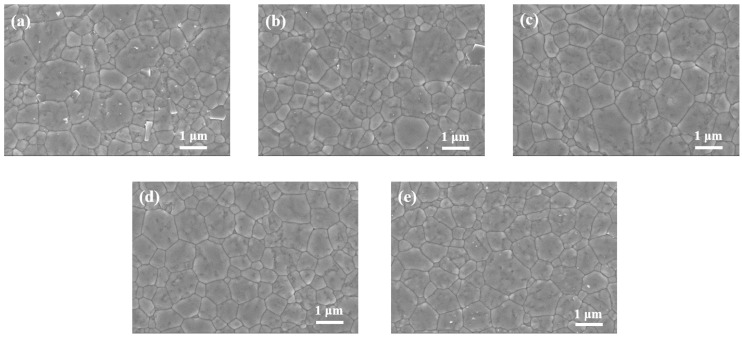
SEM images of the samples with (**a**) 1, (**b**) 3, (**c**) 5, (**d**) 7, amd (**e**) 9 at.% Er.

Figure 4a–e illustrate the SEM images of the Er:Y_2_O_3_ transparent ceramics doped with various Er concentrations. All samples showed dense microstructures, and no apparent residual pores were observed, which is consistent with the results of relative density (see Table 1). In addition, the average grain sizes of the present specimens (i.e., ~1 μm) are much finer compared to those of the previously reported Er:Y_2_O_3_ transparent ceramics fabricated by the pressureless sintering method (i.e., ~12.5 μm) [18]. Due to the mechanical pressure can effectively assist the densification of yttria so that the hot-pressing process can be carried out at much lower temperatures (e.g., 1600 °C) compared to the pressureless sintering route (e.g., ≥1800 °C), which can suppress the grain growth of Y_2_O_3_.

The grain size, microhardness, and fracture toughness of the samples are listed in Table 2. With an increase in the Er concentration from 1 to 9 at.%, the grain size slightly decreased from 0.85 to 0.76 µm, in turn, the microhardness increased from 8.51 to 8.63 GPa. It is well known that grain boundaries can block the dislocations generated by indenters, as a result, the microhardness of ceramic materials typically increases as the grain size decreases [25]. Meanwhile, the fracture toughness levels (i.e., ~1.03 MPa·m^1/2^) were not strongly affected by the doping concentration. The microhardness and fracture toughness of the present submicron-grained samples are higher compared to the large-grained Er:Y_2_O_3_ transparent ceramics by pressureless sintering (~12.5 μm in grain size, 8.05 GPa, and 0.99 MPa·m^1/2^) [18] owing to the much finer microstructures of the hot pressed samples.

**Figure 5:**
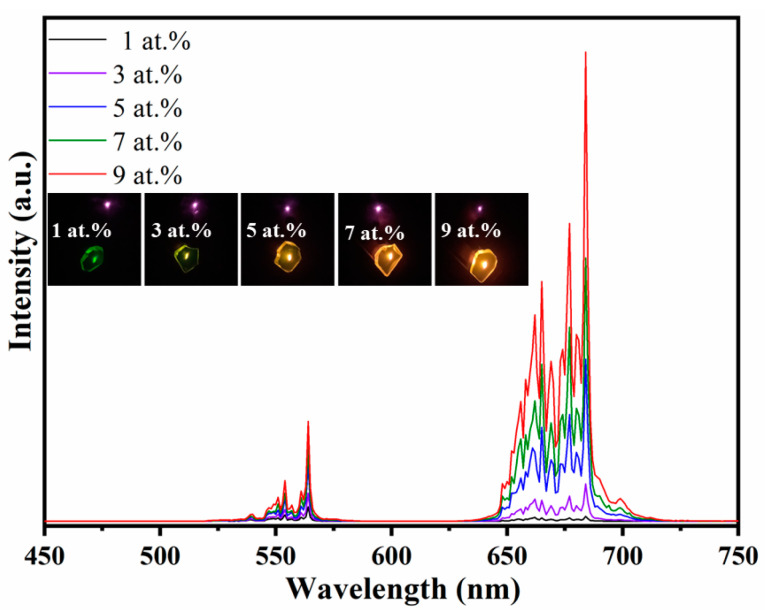
Room temperature up-conversion luminescence spectra of the samples excited by 980 nm laser, the insert shows the digital photographs of the samples under excitation.

**Figure 6:**
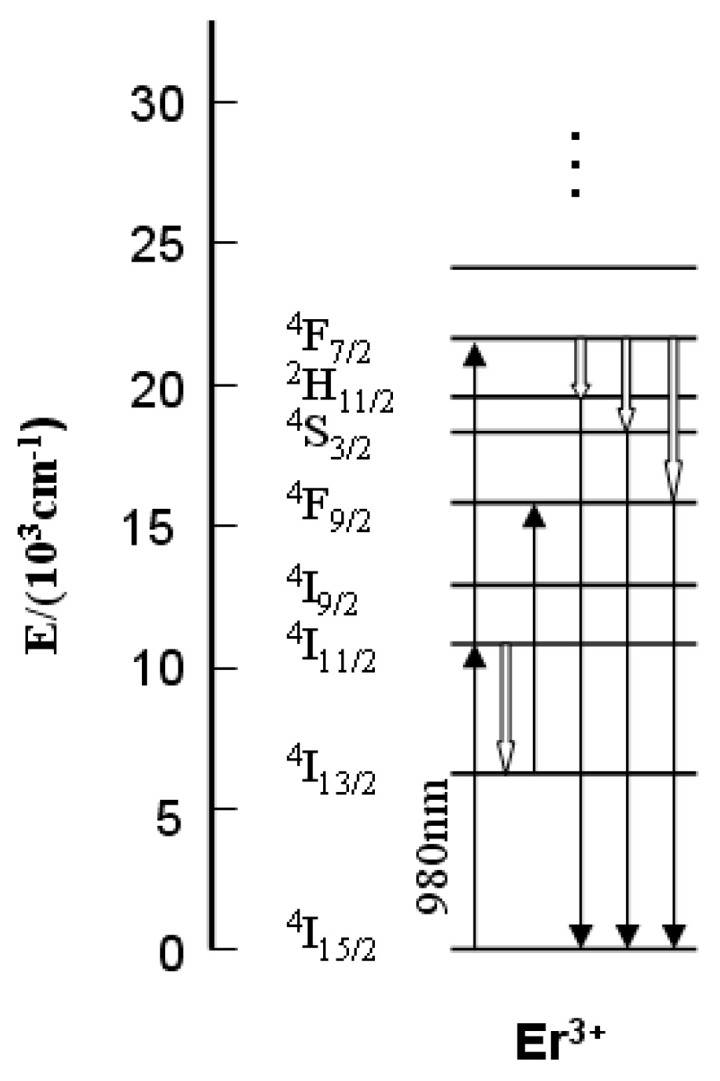
The up-conversion mechanism of Er:Y_2_O_3_ transparent ceramics under the excitation of 980 nm diode laser.

To study the luminescent properties, the room temperature up-conversion luminescence spectra of the samples were measured under 980 nm excitation of a LD. As shown in Figure 5, all samples exhibited strong up-conversion luminescence in the visible region, which centered at 563 (green, ^4^S_3/2_ → ^4^I_15/2_) and 684 nm (red, ^4^F_9/2_ → ^4^I_15/2_). The up-conversion luminescence process occurs as follows (see Figure 6):

The green emission is generated by a two-step excitation process.

^4^I_15/2_ → ^4^I_11/2_ (ground state absorption)
^4^I_11/2_ → ^4^F_7/2_ (excited stated absorption)
^4^F_7/2_ → ^2^H_11/2_/^4^S_3/2_ (multiphonon relaxation)
^2^H_11/2_/^4^S_3/2_ → ^4^I_15/2_ (radiative relaxation)

The red emission is generated by a process including multiphonon relaxation and excited state absorption.

^4^I_15/2_ → ^4^I_11/2_ (ground state absorption)
^4^I_11/2_ → ^4^I_13/2_ (multiphonon relaxation)
^4^I_13/2_ → ^4^F_9/2_ (excited state absorption)
^4^F_9/2_ → ^4^I_15/2_ (radiative relaxation)

**Figure 7:**
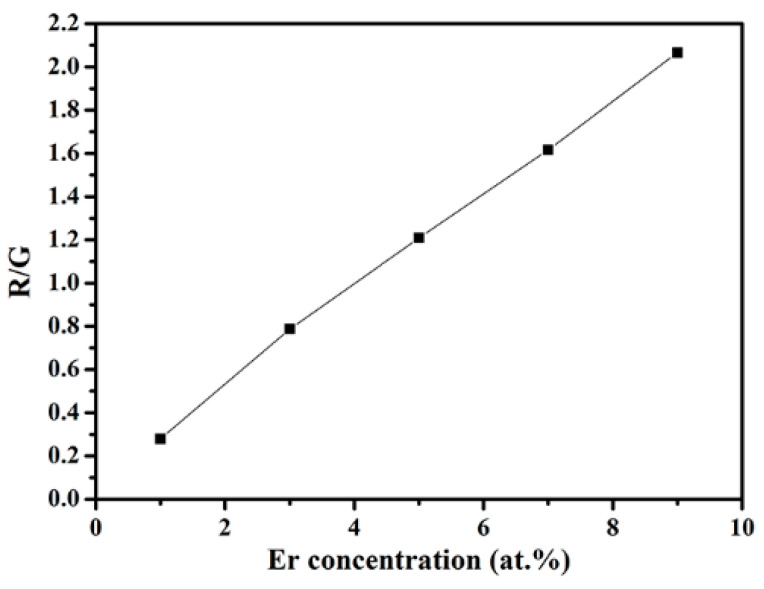
Intensity ratio of the red and green (R/G) emissions of the samples as a function of Er concentration.

The intensities of these two up-conversion luminescence emissions changed with the variation of Er-doping concentration. In general, both of them became stronger as the Er concentration increased from 1 to 9 at.%, while the red light increased much more compared to the green one and the intensity ratio of the red and green emissions gradually increased from 0.3 to 2.1 (see Figure 7). The corresponding mechanism is that an increase in the concentration of the luminescent centers (i.e., Er^3+^) has a tendency to markedly enhance the cross-relaxation effect of ^4^F_7/2_ → ^4^F_9/2_ and ^4^I_11/2_ → ^4^I_13/2_, which increases the Er^3+^ population in ^4^F_9/2_ level of the red emission [15]. The insert of Figure 5 shows the digital photographs of the samples under 980 nm excitation, clearly, as the Er doping concentration increased from 1 to 9 at.%, the emission color gradually changed from green to yellow.

**Figure 8:**
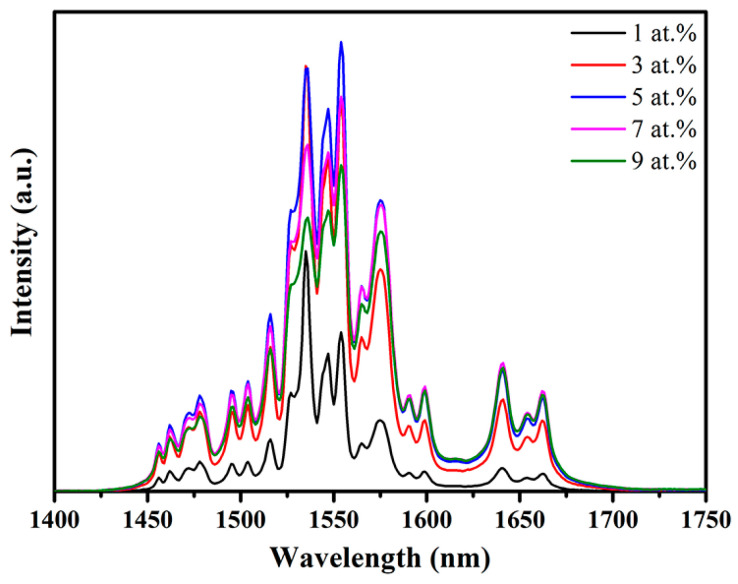
Down-conversion emission spectra of the Er:Y_2_O_3_ ceramics in the region of 1400–1750 nm.

In addition to the up-conversion luminescence, the down-conversion emission properties of the samples were also studied. Figure 8 shows the emission spectra of the specimens over the wavelength region of 1400–1750 nm under 980 nm excitation. Strong emission bands were observed at 1552, 1573, 1639, and 1661 nm, which were attributed to the ^4^I_13/2_ → ^4^I_15/2_ transition [26]. Interestingly, the doping concentration dependence of the down-conversion luminescence was different from that of the up-conversion emission. The down-conversion intensity increased as the Er concentration increased to 5 at.%, above which the intensity decreased. According to the literature, if the Er^3+^ concentration is too high (e.g., 5 at.%), the lifetimes of both ^4^I_11/2_ and ^4^I_13/2_ states will be reduced, leading to a decrease in the population of ^4^I_13/2_ level, thereby decreasing the emission intensity [27].

**Figure 9:**
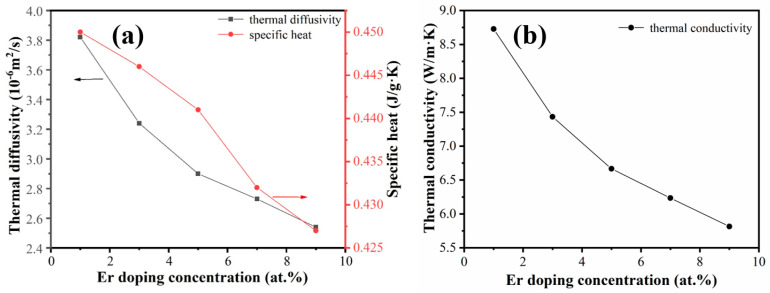
(**a**) Thermal diffusivity and specific heat and (**b**) thermal conductivity of the Er:Y_2_O_3_ transparent ceramics with various Er concentrations.

The variations of the thermal diffusivity and specific heat of the Er:Y_2_O_3_ ceramic samples as a function of the Er concentration at room temperature are presented in Figure 9a. As the Er^3+^ doping concentration increased from 1 at.% to 9 at.%, the thermal diffusivity of the sample decreased from 3.818 × 10^−6^ to 2.537 × 10^−6^ m^2^/s (decreased by 34%). On the other hand, the heat capacity was reduced by 5.1% (from 0.450 to 0.427 J/(g·K)), which was not strongly affected by the variation of the doping concentration. As a result, the thermal conductivity of the samples decreased from 8.72 to 5.81 W/(m·K). In the matrix of the Y_2_O_3_ ceramic, the heat transfer is dependent on lattice vibrations (phonon transport) [28]. When the Er^3+^ ions enter the lattice, structural distortion and point defects are introduced, resulting in stronger phonon scattering and a decrease in the phonon mean free path. This typically lowers the thermal conductivity of the ceramic samples. Zhu et al. [18] investigated the thermal properties of pressureless-sintered transparent Er:Y_2_O_3_ ceramic samples with concurrent addition of La_2_O_3_ and ZrO_2_ as sintering additives. The thermal conductivity levels of the pressureless-sintered transparent Er:Y_2_O_3_ ceramic samples were found to be much lower (i.e., ~5 W/(m·K)) compared to those the present samples, which is attributed to the higher sintering additive concentrations of the pressureless-sintered samples (i.e., 10 at.% La_2_O_3_ and 3 at.% ZrO_2_).

## 4. Conclusions

For the first time, highly transparent and submicro-grained Er:Y_2_O_3_ ceramics with various Er doping concentrations (1–9 at.%) were successfully fabricated by a hot-pressing method with 1 at.% ZrO_2_ as the sintering additive. Due to the relatively low sintering temperature and short holding time, submicron grain sizes were obtained, which led to better mechanical properties of the present samples compared to the pressureless sintered counterparts. The microhardness and fracture toughness of the hot pressed samples were ~8.51 GPa and ~1.03 MPa·m^1/2^, respectively. The present sample all exhibited high transmittance levels over the wavelength region of 400–2700 nm (e.g., ~74% at 600 nm, ~83% at 2700 nm). The up-conversion luminescence and the down-conversion emission properties of the Er:Y_2_O_3_ samples were both investigated, strong green and red up-conversion emissions were observed under the excitation of 980 nm diode laser. Meanwhile, under 980 nm excitation, strong down-conversion luminescence was also observed at 1400–1750 nm.

## Figures and Tables

**Table 1:** The structural parameters and densities of the samples with various Er concentrations.

Samples (at.%)	Lattice Parameters *a* = *b* = *c* (nm)	Unit Cell Volume (nm^3^)	Calculated Theoretical Density (g/cm^3^)	Bulk Density (g/cm^3^)	Relative Density (%)
1	1.06001	1.19104	5.0785	5.077 ± 0.007	99.97
3	1.05993	1.19079	5.1495	5.143 ± 0.012	99.87
5	1.05989	1.19066	5.2201	5.212 ± 0.005	99.84
7	1.05958	1.18960	5.2947	5.286 ± 0.010	99.84
9	1.05943	1.18910	5.3670	5.361 ± 0.006	99.88

**Table 2:** Grain size, microhardness, and fracture toughness of the samples with various Er concentrations.

Er Concentration (at.%)	Grain Size (μm)	Microhardness (GPa)	Fracture Toughness (MPa·m^1/2^)
1	0.85 ± 0.41	8.51 ± 0.03	1.03 ± 0.02
3	0.81 ± 0.37	8.53 ± 0.05	1.02 ± 0.01
5	0.82 ± 0.38	8.57 ± 0.14	1.03 ± 0.03
7	0.77 ± 0.34	8.59 ± 0.09	1.04 ± 0.01
9	0.76 ± 0.32	8.63 ± 0.08	1.05 ± 0.01

## Data Availability

The data presented in this study are available on request from the corresponding author.
